# Assembly of the outer retina in the absence of GABA synthesis in horizontal cells

**DOI:** 10.1186/1749-8104-5-15

**Published:** 2010-06-18

**Authors:** Timm Schubert, Rachel M Huckfeldt, Edward Parker, John E Campbell, Rachel OL Wong

**Affiliations:** 1 Department of Biological Structure, University of Washington, School of Medicine, 1959 NE Pacific St, Seattle, WA 98195, USA; 2 Werner Reichardt Centre for Integrative Neuroscience (CIN), Institute for Ophthalmic Research, University of Tuebingen, Roentgenweg 11, 72076 Tuebingen, Germany; 3 Department of Ophthalmology, University of Washington, School of Medicine, 1959 NE Pacific St, Seattle, WA 98195, USA

## Abstract

**Background:**

The inhibitory neurotransmitter gamma-amino-butyric acid (GABA) not only modulates excitability in the mature nervous system but also regulates neuronal differentiation and circuit development. Horizontal cells, a subset of interneurons in the outer retina, are transiently GABAergic during the period of cone photoreceptor synaptogenesis. In rodents, both horizontal cells and cone axonal terminals express GABA_A _receptors. To explore the possibility that transient GABA expression in mouse neonatal horizontal cells influences the structural development of synaptic connectivity in the outer retina, we examined a mutant in which expression of GAD67, the major synthesizing enzyme for GABA, is selectively knocked out in the retina.

**Results:**

Immunocytochemistry and electron microscopy revealed that the assembly of triad synapses involving cone axonal pedicles and the dendrites of horizontal and bipolar cells is unaffected in the mutant retina. Moreover, loss of GABA synthesis in the outer retina did not perturb the spatial distributions and cell densities of cones and horizontal cells. However, there were some structural alterations at the cellular level: the average size of horizontal cell dendritic clusters was larger in the mutant, and there was also a small but significant increase in cone photoreceptor pedicle area. Moreover, metabotropic glutamate receptor 6 (mGluR6) receptors on the dendrites of ON bipolar cells occupied a slightly larger proportion of the cone pedicle in the mutant.

**Conclusions:**

Together, our analysis shows that transient GABA synthesis in horizontal cells is not critical for synapse assembly and axonal and dendritic lamination in the outer retina. However, pre- and postsynaptic structures are somewhat enlarged in the absence of GABA in the developing outer retina, providing for a modest increase in potential contact area between cone photoreceptors and their targets. These findings differ from previous results in which pharmacological blockade of GABA_A _receptors in the neonatal rabbit retina caused a reduction in cone numbers and led to a grossly disorganized outer retina.

## Background

In addition to its essential role in the mature nervous system, the inhibitory neurotransmitter gamma-amino-butyric acid (GABA) has been shown to regulate many aspects of neuronal development [1,2], including cell proliferation, migration [3], morphogenesis [4], and circuit assembly and refinement [5,6]. Indeed, the lack of GABA synthesis in cortical interneurons decreases the number of synaptic boutons formed onto the somata of their postsynaptic targets, the pyramidal cells [5]. Some neurons, however, also receive presynaptic GABAergic input from the same postsynaptic cells they innervate [7]. But, as yet, it is not known whether perturbation of GABA synthesis in postsynaptic cells also affects the development of their connectivity with their presynaptic partners. We thus investigated this possibility in a retinal circuit in which GABA is transiently expressed during synaptogenesis by a subset of interneurons that are postsynaptic to photoreceptors.

In the outer retina of vertebrates, horizontal cell and bipolar cell dendrites are contacted by cone photoreceptors, forming synaptic 'triads' that are stereotypically arranged in a single lamina, the outer plexiform layer (OPL) [8]. Horizontal cells receive glutamatergic synaptic input from cone photoreceptors [9] and modulate photoreceptor transmission through feedback mechanisms [10,11]. Mammalian cone terminals express GABA_A _receptors [12,13] but whether activation of these receptors shapes visual responses in the mature retina is still debated [14]. This uncertainty is partially due to species variability in GABA expression by adult horizontal cells [15]. Moreover, when GABA-imunoreactivity is detected, not all horizontal cells are GABAergic across the entire retina [16]. However, horizontal cells consistently express GABA during neonatal development across mammals [17], raising the possibility that outer retinal development may be influenced by GABA.

Rodent and rabbit horizontal cells express GABA and its synthetic enzyme glutamic acid decarboxylase (GAD) during a narrow window of postnatal development, but not at maturity [18-23]. These interneurons also possess a mechanism for GABA release. VGAT, the vesicular inhibitory amino acid transporter, is expressed in the dendritic and axonal processes of horizontal cells, and it is present in the outer retina as early as birth [24-26]. In rabbit cone photoreceptor terminals, the expression of GABA_A _receptors is transient, coincident with the period of GABA synthesis by horizontal cells [27]. Correspondingly, *in vitro *application of GABA results in calcium influx in neonatal but not adult cone terminals [28]. *In vitro *treatment of rabbit retinal explants with GABA_A _receptor antagonists resulted in fewer cones [29], implicating a potential role for GABA in shaping cone photoreceptor development *in vivo*. Furthermore, because mouse horizontal cells themselves express functional GABA_A _receptors [30], it is possible that perturbation of GABA synthesis leads to abnormal development of these interneurons. Additionally, because GABA is expressed by horizontal cells during the period when bipolar cell dendrites develop and invaginate into cone pedicles and bipolar cell dendrites have GABA_A _receptors [8,31,32], it is possible that bipolar-photoreceptor synapses may be altered in the absence of GABA in the outer retina. We thus employed a genetic strategy to selectively block GABA synthesis *in vivo *in the mouse retina and then addressed whether synaptic development between horizontal cells, cone photoreceptors, and bipolar cells is altered.

## Results

### Developing mouse horizontal cells transiently express a single isoform of GAD

**Figure 1:**
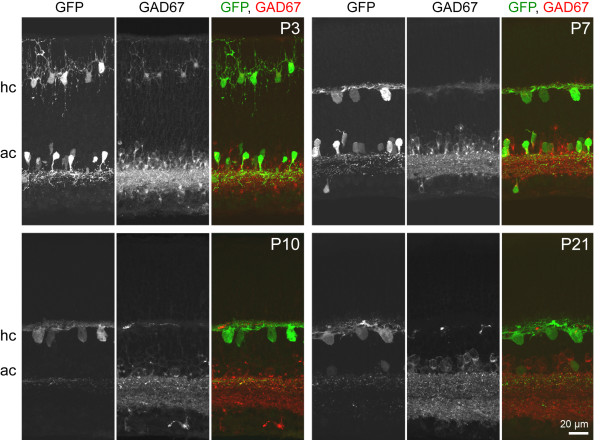
**Horizontal cells transiently express GAD67 during early postnatal development**. Retinal cross-sections from the *G42 *mouse in which horizontal cells (hc) and amacrine cells (ac) expressing GFP were immunolabeled for GAD67 during early postnatal development (P3 to P10) and at structural maturity (P21).

**Figure 2:**
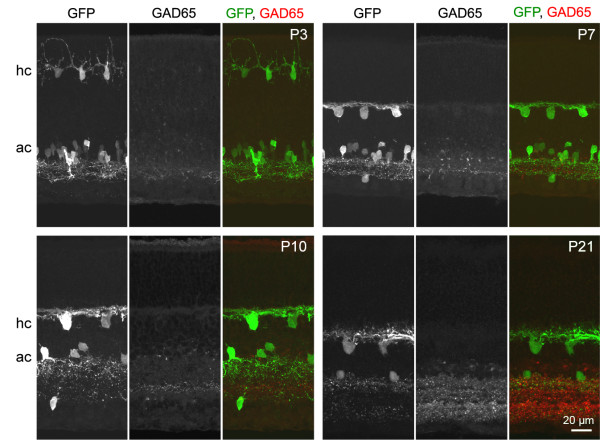
**Horizontal cells do not express GAD65 at any stage of postnatal development**. Retinal cross-sections from neonatal *G42 *mice immunolabeled for GAD65. hc, horizontal cells; ac, amacrine cells.

By using antibodies that distinguish GAD65 and GAD67, we determined which GAD isoform is transiently expressed by horizontal cells in the neonatal mouse retina [18]. We identified horizontal cells throughout development using the *G42 *transgenic mouse line in which these cells, and amacrine cells, express green fluorescent protein (GFP) [33,34]. At postnatal day 3 (P3) GAD67 expression was evident in both horizontal cells and amacrine cells (Figure 1). This pattern persisted at P7, but by P10, GAD67 expression was diminished in horizontal cells, although expression remained high in amacrine cells. Horizontal cells lacked GAD65 at all ages studied (Figure 2). In contrast, weak GAD65 staining of amacrine cells was present at P3 and increased in intensity by P21. Horizontal cells in the mouse retina thus transiently express a single isoform of the GABA synthetic enzyme, GAD67, which is down-regulated prior to eye-opening (around P14).

### GABA synthesis is abolished in Cre-expressing regions of the neonatal *αPax6-Cre:Gad1*^*lox/lox *^retina

To prevent GABA expression in the horizontal cells, we selectively inactivated both alleles of *Gad1*, the gene encoding GAD67 [35], in these cells. Because *Gad1 *knockout mice die at birth [36,37], we utilized a conditional *Gad1 *knockout line (*Gad1^lox/lox^*) [5] to obtain retina-specific *Gad1 *excision after crossing with the *αPax6-Cre *transgenic line [38]. Cre activity in retinal progenitor cells of *αPax6-Cre *mice is present by embryonic day 10.5 [38], prior to horizontal cell differentiation [39]. Thus, this Cre-expressing line is ideal for eliminating GABA synthesis from the outset of horizontal cell development. Offspring of the *αPax6-Cre:Gad1*^*lox/lox *^cross will be referred to as mutant mice.

**Figure 3:**
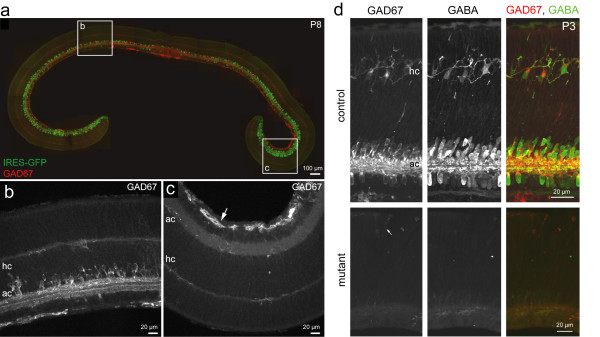
**Pattern of GAD67 expression in *αPax6-Cre:Gad1*^*lox/lox *^(mutant) retina**. **(a) **The spatial distribution of Cre recombinase expression across the retina parallels the pattern of GFP expression in amacrine cells. Cre expression is present in all cells of the embryonic retina, but several days after birth becomes restricted to subpopulations of amacrine cells [63]. At P8, GFP is expressed by relatively few cells in central retina (box, b) and a larger number of amacrine cells in the peripheral retina (box, c). Staining for GAD67 follows an inverse pattern in this mutant retina. **(b) **Numerous GAD67-immunopositive amacrine cells near the center of the mutant retina. hc, horizontal cells; ac, amacrine cells. **(c) **GAD67 immunoreactivity is largely absent in the retinal periphery of the mutant retina. Immunofluorescence in this image (arrow) marks blood vessels. There is no GAD67 immunoreactivity at retinal depths where amacrine cells (ac) and horizontal cells (hc) are located. **(d) **In the wild-type region of a P3 mutant retina, GAD67-immunopositive horizontal cells (hc) also contain GABA. In knockout regions of this retina, GAD67 and GABA immunolabeling are absent. Arrow (mutant) indicates immunoreactivity in some blood vessels.

Because Cre-recombinase is not expressed within a dorsal-ventral wedge in the mutant mouse, we limited our analysis to regions of the retina where Cre-recombination is expected to occur (that is, nasal-temporal periphery). In these regions, loss of GAD67 expression was identified in two ways. First, Cre-expressing regions were marked by the presence of GFP expression because the *αPax6-Cre *construct contains an IRES-GFP reporter cassette [38]. GFP expression in the mutant retina was apparent from birth through juvenile stages in neurons with a spatial organization consistent with that of amacrine cells (Figure 3a). GFP expression in amacrine cells could thus be used as a reporter of Cre activity within that field of view. Second, loss of GAD67 expression was directly confirmed by immunostaining for GAD67 (Figure 3b, c). Figure 3d demonstrates that a lack of GAD67 is accompanied by a loss of GABA. We did not observe expression of GAD65 in horizontal cells in any region of the mutant retina at the neonatal ages examined (data not shown).

### Lamination at the outer plexiform layer is not grossly disrupted in *Gad1 *mutant mice

**Figure 4:**
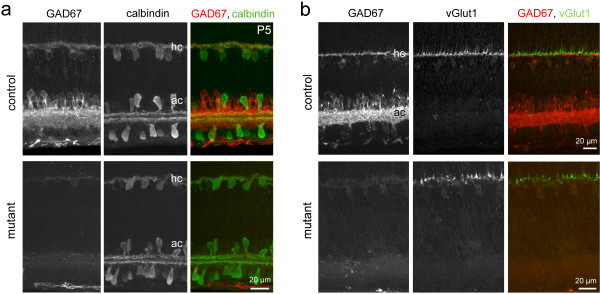
**Horizontal cell and photoreceptor lamination are not grossly disrupted in the absence of GABA in the outer retina**. **(a) **Horizontal cells from a P5 mutant retina immunolabeled for calbindin. In both littermate control and mutant retinas, the processes of these cells are stratified within the outer retina. **(b) **Photoreceptor terminals in a P5 mutant retina immunostained for vesicular glutamate transporter 1 (vGluT1). The terminals are stratified in both littermate control and mutant retinas. The slight irregularity of the mutant vGlut1 lamination is not unusual at this age and can be observed in the wild-type retina as well. hc, horizontal cells; ac, amacrine cells.

**Figure 5:**
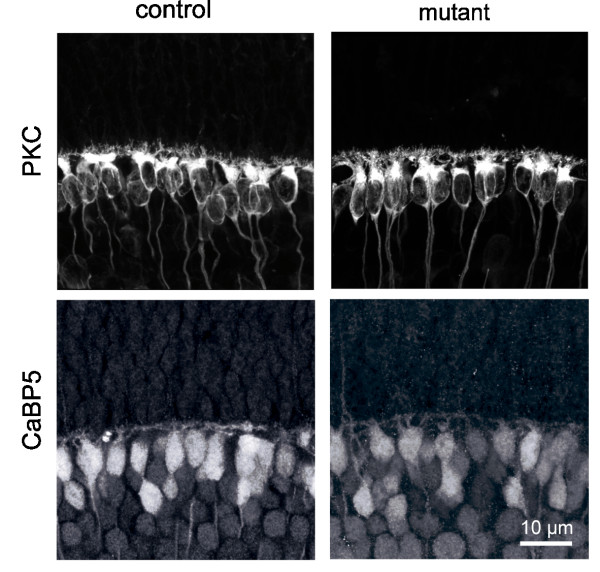
**Bipolar cell dendrites are stratified in the absence of GABA synthesis by horizontal cells**. P10 rod bipolar cells immunolabeled for PKC and cone bipolar cells immunolabeled for CaBP5 have stratified dendritic arbors in the mutant retina.

To assess the potential role for transient GABA synthesis by horizontal cells in the overall developmental organization of the outer retina, we examined the lamination of the dendrites of horizontal cells and the axon terminals of the photoreceptors. Horizontal cells undergo a transformation from a radial to a lateral morphology during early postnatal development, and their adult-like dendritic lamination is first apparent at around P5 [18,34]. This progression was not delayed in the mutant retina, compared to littermate controls (*Gad1*^*lox/lox*^). By P5, horizontal cells in both control and mutant retinas had assumed a laminar morphology (Figure 4a) that is maintained at maturity (not shown). Cone photoreceptors also did not exhibit any gross lamination deficits in the mutant retina. When P5 cone axonal terminals were identified with vesicular glutamate transporter 1 (vGlut1) immunolabeling [40], they were appropriately targeted to the OPL in the mutant retina (Figure 4b). In addition, the targeting and lamination of bipolar cell dendrites to the OPL was not disrupted in the absence of GABA. At P10, the dendrites of protein kinase C (PKC)-positive rod bipolar cells and calcium binding protein 5 (CaBP5)-positive cone bipolar cells ramified in the OPL in both mutant and control retinas (Figure 5). Thus, in contrast to previous reports [29,41], OPL lamination proceeded normally in the absence of GABA synthesis by horizontal cells.

### Density and distribution of horizontal cells and cones are unaffected in the *Gad1 *mutant retina

**Figure 6:**
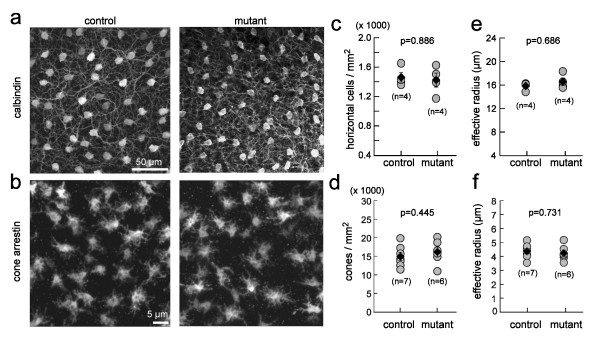
**Density and distribution of horizontal cells and cones are normal in the mutant retina**. **(a,b) **Populations of horizontal cells (a) and cone pedicles (b) immunostained for calbindin and cone-arrestin, respectively, for P11 retinas. **(c,d) **Plots of the density of horizontal cells and cones for littermate controls and mutant retinas. **(e,f) **Comparison of effective radii for horizontal cells and cones in littermate controls and mutant retinas. (c-f) n = number of retinas; filled diamond and error bar = mean and standard error.

Although transient GABA expression in horizontal cells does not affect the lamination of the OPL, it remained possible that trophic actions of GABA could influence other organizational aspects of the outer retina, such as cell density and spatial distributions of the somata of cones and horizontal cells. The populations of horizontal cells and cones were thus immunolabeled using anti-calbindin and anti-cone-arrestin, respectively (Figure 6a, b). Quantitative analysis showed that the densities of both horizontal cells (Figure 6c) and cones (Figure 6d) were unaltered in the mutant retina.

We also determined whether the mosaic arrangement of the cell bodies of horizontal cells and the average distance between cone photoreceptors were altered in knockout regions of the mutant retina. To do so, we obtained the density recovery profile for each cell population and calculated the effective radius. The effective radius quantifies the extent of the region of decreased probability of encountering nearby somata of the same cell type and is therefore a measure for the regularity of cell body distribution [34,42]. Neither the effective radii of the horizontal cell population (Figure 6e) nor that of the cone population (Figure 6f) was statistically different between mutant and control retinas.

### Horizontal cell dendritic terminal cluster and cone pedicle areas are increased in *Gad1 *mutant retina

**Figure 7:**
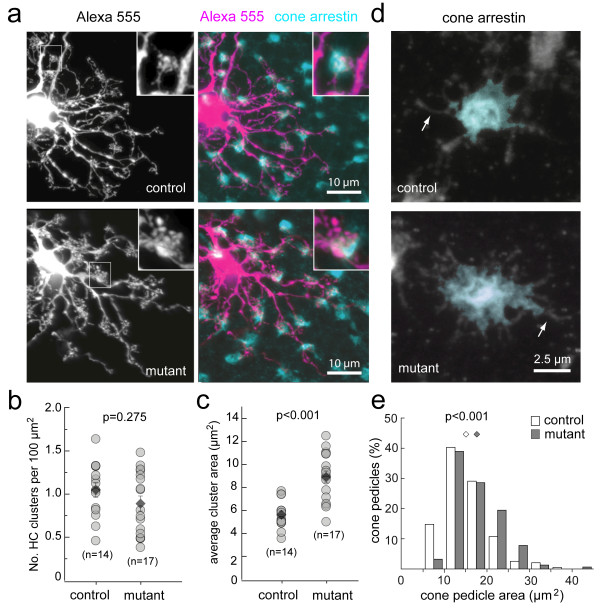
**Horizontal cell dendritic cluster area and cone pedicle size are significantly increased in the mutant retina**. **(a) **Examples of horizontal cells in P10 littermate control and mutant retinas that were intracellularly dye-filled with Alexa Fluor 555. Mutant retinas were immunostained for GAD67 to determine whether cells were located in the wild-type or knockout regions (not shown). Insets show dendritic terminal clusters that overlay cone pedicles (revealed by immunostaining for cone-arrestin) at their sites of contact. **(b,c) **Quantification of horizontal cell (HC) terminal cluster density (number of clusters per 100 μm^2 ^dendritic field area) and average cluster area per cell. n = number of retinas. Filled diamond and error bar = mean and standard error. **(d) **High magnification examples of the maximum intensity projections of an image stack through a cone pedicle in a littermate control retina and a pedicle in the mutant retina. Colored regions within the cone pedicles mark the cone pedicle 'area' determined from their respective labeled-fields (see Materials and methods). Arrows indicate an example of filopodia that decorate the base of the cone pedicles. **(e) **Distributions of cone pedicle areas in littermate control and mutant retinas. Diamonds indicate means of the distributions: mutant (17.15 ± 0.46 μm^2^), n = 154 pedicles, n = 4 animals; control (15.20 ± 0.38 μm^2^), n = 196 pedicles, 5 animals.

We quantified the number of dendritic terminal clusters formed by individual horizontal cells. Varicosities within these terminal clusters have been shown previously to invaginate cone pedicles and thus represent postsynaptic sites on the horizontal cell dendrites [43]. To visualize the dendritic terminal clusters of individual horizontal cells, we intracellularly dye-filled these cells in live retina. Figure 7a shows examples of the dendritic arbors of dye-filled horizontal cells in mutant and littermate control retinas at P10, when GAD67 expression is normally just down-regulated in the horizontal cells. The average dendritic field sizes of the horizontal cells in mutant (4,500 ± 282 μm^2^, n = 30 cells) retinas were similar (*P *= 0.644) to that of controls (4,760 ± 512 μm^2^, n = 25 cells). Moreover, there was no difference between the densities of terminal clusters formed by horizontal cell dendrites (number of clusters per dendritic field area) in the mutant and control (Figure 7b). Because cone density is unchanged in the mutant retina, this observation suggests that horizontal cells in the mutant mice are likely to contact the same average number of cones as in control retinas. Comparison of the average sizes of individual terminal clusters, however, revealed a statistically significant increase (*P *< 0.001) in this measure in mutant mice (Figure 7c). The mean areas of the clusters were 5.65 ± 0.32 μm^2^ (n = 14 cells) for control and 8.90 ± 0.51 μm^2^ (n = 17 cells) for mutant retinas.

We then wondered whether the increase in horizontal cell dendritic cluster size was paralleled by an increase in cone pedicle sizes. Individual cone pedicle areas were thus measured in cone-arrestin labeled retinas (see Materials and methods). Figure 7d shows the region of the cone pedicles (highlighted in color) that represent the pedicle area. Filopodia (arrows) extending from the cone pedicles were not included in such measurements. Although there is a broad distribution of pedicle areas across retinas and even within a single field of view, the mean area was significantly increased (*P *< 0.001) in the mutant retinas (17.15 ± 0.46 μm^2^, n = 154 pedicles, n = 4 animals) compared to control (15.20 ± 0.38 μm^2^, n = 196 pedicles, n = 5 animals) retinas (Figure 7e). Thus, horizontal cell dendritic clusters and cone pedicles are, on average, larger in mutant mice.

### Outer retinal synapse components in the *Gad1 *mutant retina appear normal

**Figure 8:**
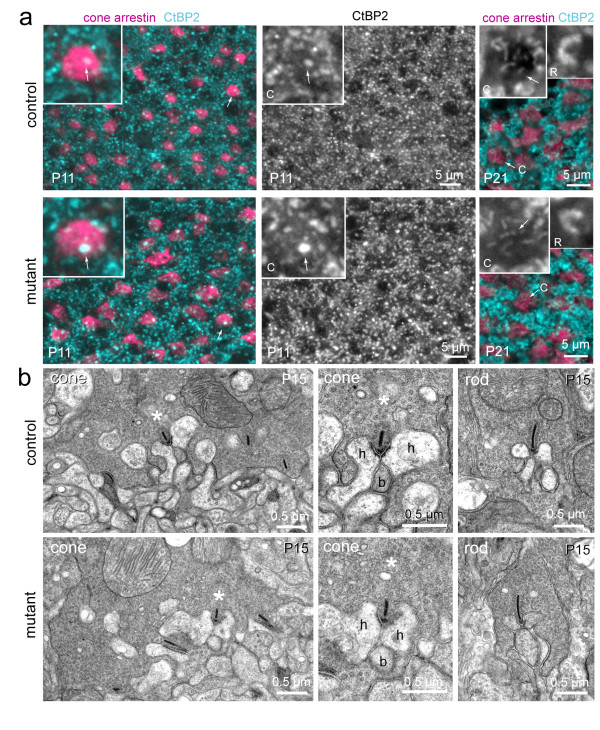
**Ribbons are present and localized to synaptic sites in the mutant retina**. **(a) **Immunostaining for cone pedicles (cone-arrestin) and ribbons (C-terminus binding protein 2 (CtBP2)) in littermate controls and knockout regions of the mutant retina. Insets show higher magnification of cone pedicles marked by arrows in merged images. Arrows in insets indicate bright CtBP2 immunoreactivity - note more rounded structures at P11 compared to ribbon-like structures at P21 for cones. C, cone ribbons. Additionally, higher magnifications of CtBP2 immunostained horseshoe-like ribbons in rod terminals (R) are shown for the P21 retinas. **(b) **Examples of the ultrastructure of a cone and rod pedicles in control and mutant retinas showing localization of ribbons at synaptic triads. For each of the cone examples, the asterisk indicates the same triad shown in the left and middle columns (h, horizontal cell process; b, bipolar cell dendrite).

To investigate whether the increase in horizontal cell and photoreceptor terminal sizes in the mutant retina reflect changes in synapse structure, mutant and control littermates were immunostained for cone-arrestin and C-terminal binding protein 2 (CtBP2) to co-label cone pedicles and synaptic ribbons, respectively. Figure 8a shows that ribbon-like structures were readily found within cone pedicles in both the mutant retina and littermate controls. To obtain a higher resolution image of the ribbon synapses formed by photoreceptors onto horizontal cells, electron microscopy was carried out on mutant and control retinas at P15, when ribbons are readily identified. Both cones and rods made classic triad ribbon synapses with horizontal cells and bipolar cells (Figure 8b), suggesting that lack of GABA synthesis in horizontal cells during the period of photoreceptor synaptogenesis does not affect the fine structure of this synapse.

**Figure 9:**
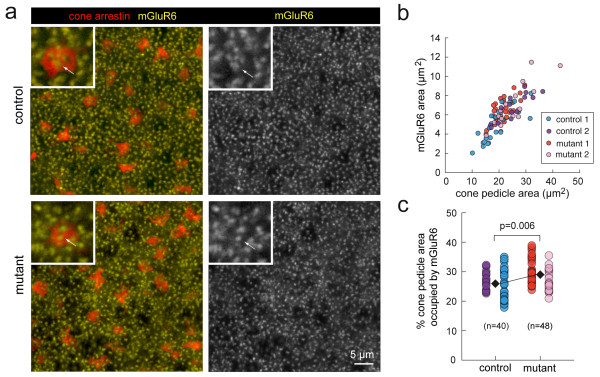
**Total postsynaptic metabotropic glutamate receptor area within cone pedicles is increased in the mutant retina**. **(a) **Co-labeling of cone pedicles (cone-arrestin) and metabotropic glutamate receptor 6 (mGluR6) on ON bipolar cell dendritic tips at P11. Insets show higher magnification images of the cone pedicles and mGluR6 receptor clusters (arrows). mGluR6 immunoreactivity outside the cone pedicles is associated with rod photoreceptor terminals. **(b) **Within each cone pedicle, a threshold was applied to the mGluR6 signal and the total area of pixels with intensities above the threshold is plotted against pedicle area. **(c) **For each cone, the total pixel area corresponding to mGluR6 signal above threshold is presented as a percentage of the area of that cone pedicle. n = number of pedicles analyzed, two animals each for control (three fields) and mutant retinas (three fields).

However, it remained possible that glutamate receptor distributions on bipolar cell dendrites that contact cone pedicles may be altered. We thus co-immunolabeled mutant and control retinas for cone-arrestin and for mGluR6, the metabotropic glutamate receptor on dendritic tips of the invaginating ON bipolar cells (Figure 9). mGluR6 receptor clusters overlaid cone pedicles of the mutant and control retinas (Figure 9a). Because it is difficult to count individual receptor clusters confidently due to the resolution of light microscopy, we obtained an estimate of receptor distribution by quantifying the total area occupied by mGluR6 receptor clusters within each cone pedicle (see Materials and methods). Plots of the absolute areas (Figure 9b) for pedicles in the mutant and control retinas demonstrate that, in general, larger pedicles have greater total receptor areas. The average total mGluR6 area within a cone pedicle for wild-type retina was 5.58 ± 0.29 μm^2 ^(n = 40 pedicles, 2 animals) and for the mutant, 6.59 ± 0.23 μm^2 ^(n = 48 pedicles, 2 animals) (*P *< 0.016). We normalized for cone pedicle size by calculating the percentage of pedicle area occupied by mGluR6 receptor clusters, and found that there was a small, although significant, increase in this measure in the mutant retina (29.00 ± 0.64%), compared to controls (25.90 ± 0.74%) (Figure 9c).

## Discussion

### GABAergic regulation of neuronal differentiation

In past studies, treatment with GABA receptor antagonists disrupted the migration of cortical pyramidal cells, cortical interneurons, and hippocampal neurons [44,45]. This developmental influence of GABA has been attributed to its depolarizing effect on immature neurons. Prior to expression of the K-Cl cotransporter (KCC2), the Na-K-Cl cotransporter (NKCC) accumulates chloride ions, resulting in membrane depolarization following the activation of GABA_A _receptors. Depolarization activates voltage-gated calcium channels and consequently has the potential to activate numerous calcium-dependent signaling cascades. GABA-evoked depolarization has been shown to regulate the exit of neural progenitors from the cell cycle, and GABA may also influence the survival of differentiated neurons (reviewed in [2]). In the rat retina, KCC2 is not expressed by horizontal cells and bipolar cell dendrites until P7, well after the initial stages of OPL assembly, and it is not detected at all in photoreceptor terminals [46]. This relatively late onset of KCC2 expression and the finding that GABA application causes calcium influx into cone terminals in rabbit neonatal retinal explants [28] led to the hypothesis that GABA may exert a trophic role during the early development of the outer retina. Indeed, the number of cones in the neonatal rabbit retina is reduced following pharmacological blockade of GABA_A _receptors in the retina maintained in culture over several days [29,47]. In contrast to these previous studies, we did not find any migratory defects in the mutant retinas; horizontal cells attained an appropriate laminar position in the outer retina in the mutant retina. Moreover, the lack of GABA synthesis in the horizontal cells in the mutant retina did not affect the density of horizontal cells or cone photoreceptors.

GABA has also been shown to have a neuritogenic effect. For example, treatment with GABA increases neurite number and morphological complexity, whereas application of GABA receptor antagonists or loss of functional GABA_A _receptors results in simplification of neurite branching [4,48-50]. We found that the dendrites of horizontal cells as well as the axonal terminals of the cone photoreceptors were, however, appropriately targeted and stratified in the outer retina. Overall, the dendritic morphology of horizontal cells in the mutant retina also resembled those in the wild-type retina, and the dendrites occupied territories of similar size. However, there were morphological changes at the dendritic terminals of the horizontal cells. Horizontal cells in the mutant retina possessed larger clusters of postsynaptic varicosities, in contrast to the smaller axonal boutons found in cortical interneurons lacking GAD67 [5].

Our findings differ from observations made following intraocular injections of kainic acid that ablated horizontal cells as well as findings following the incubation of neonatal rabbit retinal explants with GABA_A _receptor antagonists [29,41,47]. In these earlier studies, cone terminals were rarely observed in the OPL, and this synaptic plexus was severely perturbed. The lack of gross abnormalities in the OPL of the mutant retina we examined here is, however, in accord with studies of full GAD67 knockout animals. No large-scale defects were apparent in embryonic and P0 brains of GAD67 knockout animals [36], and organotypic cultures of hippocampus and cerebellum from these animals developed normally [51]. Similarly, the brains of mice deficient for both GAD65 and GAD67 lack major structural abnormalities [52], and retinal lamination is normal in a mutant that lacks *Gad2 *completely and possesses a single copy of *Gad1 *[53]. However, unlike these studies, restricting the loss of GAD67 expression to the retina enabled us to examine circuits that are assembled well after birth, and in the background of a complete knockout of *Gad1*.

One possible explanation for the different outcomes of our study and previous work on the rabbit retina is provided by Owens and Kriegstein [1], who suggested that in germline mutants, alternative pathways may compensate for the absence of GABA synthesis and preserve normal development. They proposed taurine signaling as a candidate mechanism. This cysteine derivative is present in high levels in the developing nervous system and can activate both glycine and GABA_A _receptors [54,55]. In the rat retina, taurine is present at least transiently in all neuronal types [56]. Moreover, taurine acting on glycine receptors on retinal progenitor cells increases the production of rod photoreceptors [57]. Thus, it remains possible that in the mutant retina, taurine-mediated signaling may have compensated for the absence of GABA production in horizontal cells. Such compensatory mechanisms may not be present in retinal explants treated with GABA_A _receptor antagonists. Regardless of whether there might be compensation by other signaling pathways in the mutant mice, our current findings suggest that structural assembly of the OPL does not critically depend on the expression of GABA in horizontal cells. It remains to be tested whether loss of horizontal cells altogether would evoke large structural changes in the outer retina.

### Influence of GABA on synapse development

There is increasing evidence to support a role for GABA in synapse development in the central nervous system. Stellate interneurons in mice lacking the alpha-1 subunit of the GABA_A _receptor form synapses with their targets the Purkinje cells, but the early connections are misrouted with maturation to innervate dendritic spines [58]. Thus, GABAergic transmission could help direct targeting of GABAergic axons to appropriate postsynaptic sites. More recently, a developmental role for GABA in synapse development was highlighted by findings in the visual cortex. Conditional knockout of GAD67 in basket interneurons led to sparser innervation of pyramidal neurons as well as the formation of fewer and smaller axonal terminal perisomatic boutons [5]. In these cases, both development and maturation of the presynaptic terminal of the GABAergic neuron appears perturbed.

Compared to other central nervous system circuits, the horizontal cell-cone photoreceptor synapse is unusual in its arrangement. Here, the GABAergic interneuron, the horizontal cell, is postsynaptic to the cone axon terminal that bears GABA_A _receptors. We found that knockout of GABA synthesis in horizontal cells did not alter the specialized pre- and postsynaptic triad organization of the photoreceptor, horizontal, and bipolar cell synapse. Presynaptic ribbons were localized appropriately opposite postsynaptic processes. However, we did find that the dendritic terminal clusters of horizontal cells that invaginate cone pedicles were, on average, larger in the mutant retina. The cones also exhibited a corresponding increase in their pedicle area. An increase, rather than decrease, in potential contact area between the dendrites of horizontal cells and the cones is unexpected given that past studies reveal a trophic role for GABA during development. It should be noted that the OPL is also innervated by GABAergic interplexiform cells [59]. However, in the mutant retina, these cells should have much reduced GABA synthesis since GAD67 expression was also significantly reduced in amacrine cells.

Whether the increase in cone pedicle and horizontal cell dendritic clusters reflects a functional change in the transmission from cone photoreceptors remains to be determined. To date, measuring cone transmission onto horizontal cells or bipolar cells in the mouse retina has not yet been performed in detail by electrophysiological methods, largely because such recordings are technically challenging. Thus, until physiological responses to light are fully documented for horizontal cells and bipolar cells in the wild-type mouse retina, it is not possible to comprehensively assay for potential changes in function in the OPL in the *Gad1 *mutant retina. Our current observations, however, would suggest that if present, physiological alterations in photoreceptor transmission in the mutant retina are unlikely to be severe, given the modest changes in structure we observed in the OPL.

## Methods

All experiments were carried out under the guidelines of the University of Washington Institutional Animal Care and Use Committee (IACUC).

### Mutant mice

Several transgenic mouse lines were used in this study. In the *G42 *mouse line, the *Gad1 *promoter drives GFP expression in horizontal cells [34]. In the *Gad1*^*lox/lox *^mice, exon 2 of *Gad1*, the gene encoding GAD67, is flanked by loxP sites [5]. To obtain a retina-specific excision of exon 2, this line was bred to the *αPax6-Cre *line, in which the expression of Cre-recombinase is regulated by the alpha element of the *Pax6 *promoter [38]. The excision of exon 2 in cells expressing Cre-recombinase results in a frameshift mutation of *Gad1*[5].

### Dye-filling

Tissue dissection and dye-filling of single horizontal cells with Alexa Fluor 555 hydrazide (Invitrogen, Carlsbad, California, USA) was performed as previously described [34]. In brief, isolated retinas were divided into four to eight pieces and incubated in Ames solution (pH 7.4; Sigma, St Louis, MO, USA) containing 10 μM 4',6-diamidino-2-phenylindole (DAPI; Sigma) for 90 minutes at room temperature and then mounted on black filter paper (Millipore, Billerica, MA, USA) with the ganglion cell layer up. Intracellular injections were carried out with borosilicate glass electrodes (170 to 200 MΩ, P-87, Sutter Instruments, Novato, CA, USA) and filled with 5 mM Alexa Fluor 555 hydrazide dissolved in 0.1 M TRIS buffer. DAPI-stained horizontal cell somata were identified based on their large size and their location in the inner nuclear layer (INL) in close proximity to the OPL. After impaling the cell under a 63× water-immersion objective (Zeiss Achroplan, 0.6 NA), Alexa Fluor 555 was delivered using a continuous current of -1 nA. Only one horizontal cell was injected per retinal piece. After dye-filling, the pieces of retina were fixed in 4% paraformaldehyde for 20 minutes. The tissue was then washed and stored in 0.1 M phosphate-buffered saline.

### Immunostaining

Fixed retinas were sectioned (60 μm thickness) using a vibratome. Immunolabeling was performed using antibodies directed against GAD67 (mouse monoclonal anti-GAD67, 1:1,000; MAB5406, Chemicon, Billerica, MA, USA), GAD65 (rabbit anti-GAD65, 1:1,000; AB5082, Chemicon), GABA (guinea pig anti-GABA, 1:1,000; Chemicon), calbindin (rabbit anti-calbindin, 1:1,000; Swant, Bellinzona, Switzerland), cone-arrestin (rabbit anti-cone-arrestin, 1:400; gift of C Craft [60,61]), C-terminal binding protein 2 (mouse anti-CtBP2, 1:1,000; BD Biosciences, San Jose, CA, USA), CaBP5 (rabbit anti-CaBP5, 1:400; gift of F Haeseleer and K Palczewski), PKC (rabbit anti-PKC, 1:1,000; Chemicon), mGluR6 (sheep anti-mGluR6, 1:100; gift of C Morgans) and vGluT1 (rabbit anti-vGluT1, 1:1000; Chemicon). Secondary antibodies were Alexa Fluor 488, Alexa Fluor 568, and Alexa Fluor 633 conjugates (1:1,000; Invitrogen).

### Electron microscopy

Retinas were fixed by immersion of the eyecup in 2% paraformaldehyde/2% glutaraldehyde in 0.1 M sodium cacodylate buffer, pH 7.4 for 3 hours. The tissue was then washed in buffer and further fixed in 1% osmium tetroxide in cacodylate buffer for an hour prior to *en bloc *staining with uranyl acetate (1%). Subsequently, the tissue was dehydrated in a graded ethanol series, embedded in araldite resin, sectioned and stained with lead citrate.

### Imaging

Fixed tissue was imaged using an Olympus FV1000 laser scanning microscope and an Olympus 60× oil objective (1.35 NA). The z-step (0.3 μm) was chosen according to the pixel size determined by the numerical aperture of the objective and the magnification. Each optical plane was averaged three to four times with a Kalman filter and the channels were acquired sequentially.

### Analysis and statistics

Raw image stacks were processed using MetaMorph (Molecular Devices, Downingtown, PA, USA), Amira (Mercury Computer Systems, Chelmsford, MA, USA) and Imaris (Bitplane Scientific Software, Zurich, Switzerland). To confirm the loss of GAD67 in the region whereby horizontal cells were dye-filled, we performed GAD67 immunochemistry on the retina after dye-filling. Knockout regions could also be identified for intracellular dye-filling experiments at locations where there was a high density of amacrine cells expressing GFP (typically along a dorsal-ventral wedge).

For analysis of cell mosaic characteristics, the maximum intensity projections of cone-arrestin-labeled image stacks and calbindin-labeled horizontal cell image stacks were imported into Matlab (Mathworks, Natick, MA, USA). The density of horizontal cells and cone photoreceptors as well as the effective radius of their density recovery profile were calculated using custom-written Matlab routines (JL Morgan), as previously described [34].

To determine the dendritic field size of horizontal cells, dye-filled horizontal cells were first skeletonized in three-dimension (Imaris). Their dendritic field areas were then determined as the area of a polygon that encompassed all distal dendritic tips in the two-dimensional projection of the skeletonized arbor (Metamorph; custom-written Matlab routine by D Kerschensteiner). Dendritic terminal cluster sizes of dye-injected horizontal cells were obtained by calculating the areas within the outlines of the clusters in the maximum intensity projection of the cells (Metamorph; Matlab routine by D Kerschensteiner). Because dendritic clusters are not uniform across the dendritic field (they are smaller on the distal dendrites of horizontal cells [62]), we restricted our analysis to clusters located within the proximal half of the dendritic field.

Cone pedicle sizes and mGluR6 receptor area were obtained by using the label-field function of AMIRA. Briefly, for each image stack of cone-arrestin labeling, a threshold is set manually for each cone pedicle and only pixels representing the cone pedicle throughout the image stack are kept within a 'labeled' region. The variation in brightness of staining even within a single field of view required thresholds to be set for each cone separately. Pixels outside of the 'labeled' region were assigned a value of '0' whereas pixels within were assigned a value of '1'. To isolate mGluR6 clusters within cone pedicles from those within rod spherules, the cone three-dimensional labeled-field was multiplied by the image stack of the mGluR6 channel. This step identified mGluR6 signal only within the volume of the cone pedicle. The area of each cone pedicle was represented by the area of the maximum projection of the labeled-field. This is because the cone pedicle is widest at its base where contact is made with horizontal cell and bipolar cell dendrites. The total mGluR6 area was calculated from the maximum intensity projection of the pixels within each cone terminal above a threshold manually set above the background mGluR6 immunofluorescence within that pedicle.

Wilcoxon rank-sum test (Sigma Stat; Systat Software, Chicago, IL, USA) was used to ascertain statistical significance for all measurements.

## Abbreviations

GABA: gamma-amino-butyric acid; GAD: glutamic acid decarboxylase; GFP: green fluorescent protein; KCC2: K-Cl cotransporter; mGluR6: metabotropic glutamate receptor 6; OPL: outer plexiform layer; P: postnatal day; PKC: protein kinase C; vGluT1: vesicular glutamate transporter 1.

## Competing interests

The authors declare that they have no competing interests.

## Authors' contributions

TS, RMH and ROLW planned the study, performed the experiments, analyzed the data and prepared the manuscript. JEC performed parts of the immunolabeling studies, and EP carried out the electron microscopy.
